# Btp Proteins from *Brucella abortus* Modulate the Lung Innate Immune Response to Infection by the Respiratory Route

**DOI:** 10.3389/fimmu.2017.01011

**Published:** 2017-08-24

**Authors:** Maria Soledad Hielpos, Mariana C. Ferrero, Andrea G. Fernández, Juliana Falivene, Silvia Vanzulli, Diego J. Comerci, Pablo C. Baldi

**Affiliations:** ^1^Universidad de Buenos Aires, Facultad de Farmacia y Bioquímica, Cátedra de Inmunología, Buenos Aires, Argentina; ^2^CONICET-Universidad de Buenos Aires, Instituto de Estudios de la Inmunidad Humoral (IDEHU), Buenos Aires, Argentina; ^3^Instituto de Investigaciones Biotecnológicas (IIB, UNSAM-CONICET), San Martín, Argentina; ^4^Laboratorio de Anatomía Patológica, Instituto de Estudios Oncológicos, Academia Nacional de Medicina, Buenos Aires, Argentina

**Keywords:** *Brucella abortus*, respiratory infection, innate immunity, immunomodulation, inflammation

## Abstract

Although inhalation of infected aerosols is a frequent route for *Brucella* infection in humans, it rarely causes pulmonary clinical manifestations, suggesting a mild or nearly absent local inflammatory response. The goal of this study was to characterize the early innate immune response to intratracheal infection with *Brucella abortus* in mice and to evaluate whether it is modulated by this pathogen. After infection with 10^6^ CFU of *B. abortus*, the pulmonary bacterial burden at 7 days post-infection (p.i.) was comparable to the initial inoculum, despite an initial transient decline. *Brucella* was detected in spleen and liver as early as 1 day p.i. IL-1β and MCP-1 increased at 3 days p.i., whereas IL-12, KC, TNF-α, and IFN-γ only increased at 7 days p.i. Histological examination did not reveal peribronchial or perivascular infiltrates in infected mice. Experiments were conducted to evaluate if the limited inflammatory lung response to *B. abortus*is caused by a bacterial mechanism of TLR signaling inhibition. Whereas inoculation of *E. coli* LPS to control mice [phosphate-buffered saline (PBS)/LPS] caused lung inflammation, almost no histological changes were observed in mice preinfected intratracheally with *B. abortus* (WT/LPS). We speculated that the *Brucella* TIR-containing proteins (Btps) A and B, which impair TLR signaling *in vitro*, may be involved in this modulation. After LPS challenge, mice preinfected with the *B. abortus btpAbtpB* double mutant exhibited a stronger pulmonary polymorphonuclear infiltrate than WT/LPS mice, although milder than that of the PBS/LPS group. In addition, lungs from *B. abortus btpAbtpB*-infected mice presented a stronger inflammatory infiltrate than those infected with the WT strain, and at day 7 p.i., the pulmonary levels of KC, MCP-1, and IL-12 were higher in mice infected with the mutant. This study shows that *B. abortus* infection produces a mild proinflammatory response in murine lungs, partially due to immune modulation by its Btp proteins. This may facilitate its survival and dissemination to peripheral organs.

## Introduction

Brucellosis is a worldwide-distributed zoonotic disease caused by *Brucella* species that affects over 500,000 people annually (1). Most cases are caused by *Brucella melitensis, Brucella suis*, and *Brucella abortus*. The infection can be transmitted to humans by several ways, among which inhalation of infected aerosols is one of the most frequent. Several reports place airborne transmission as the cause of outbreaks of human brucellosis in bovine and porcine slaughterhouses, vaccine production laboratories, and rural areas (2–5). Notably, aerosols have been implicated in most cases of laboratory-acquired brucellosis, which is considered the most common laboratory-acquired infection (6). Due to its easy aerosolization, high infectivity and airborne transmission, *Brucella* species are considered potential biological weapons (1) and are classified by the CDC and NIAID as category B bioterrorism agents.

Airborne *Brucella* infection rarely causes pulmonary clinical manifestations in infected humans. In case series in which inhalation of organisms was strongly suspected as the most probable source of infection, lung pathology was extremely rare (4, 7, 8). These observations suggest that, in spite of the high infectivity of *Brucella* through inhalation, an inflammatory immune response against the pathogen is mild or nearly absent in the lungs. *Brucella* species use diverse mechanisms to evade innate immunity, some of which may contribute to a limited inflammatory response to the pathogen in the lungs. The ability of *Brucella* to impair TLR signaling may be particularly important in this regard (9, 10).

The innate immune response against *Brucella* is of great importance because it determines the course of the adaptive response (11, 12), which is key for the control of *Brucella* infection. A critical step for the initiation of the innate immune response is the detection of microbial PAMPs by TLR receptors. TLR9 has been shown to be required to eliminate *B. abortus* in infected mice (13). Similarly, mice deficient in MyD88 (the adaptor molecule for all TLRs except TLR3) are highly susceptible to *Brucella* infection (11, 13, 14). Although TLR2 does not seem to be essential for *Brucella* control in infected mice (11, 15), it has been extensively shown that signaling through TLR2 contributes to the production of proinflammatory cytokines by *Brucella*-infected phagocytic and non-phagocytic cells (16–19). The role of TLR4 remains controversial. Whereas some studies show that TLR4 is required to control *Brucella* infection (15), others do not reveal such requirement in spite of the importance of this receptor for TNF-α production (11). Although the role of TLR5 in *Brucella* infections has not been explored *in vivo*, it has been shown that *Brucella* flagellin is not recognized by human TLR5 (20).

Notably, *Brucella* expresses two proteins that impair signaling through TLR2, TLR4, TLR5, and TLR9, named *Brucella* TIR-containing proteins (Btps) A and B (9, 10). Although these proteins have been shown to reduce cytokine secretion by cells infected with *Brucella in vitro*, a potential role of Btps in the ability of *Brucella* to survive in immunocompetent hosts has not been investigated.

A previous study reported that *B. abortus* can infect Balb/c mice through aerosols and can persist at high numbers in the lungs for several weeks, suggesting that an effective pulmonary immune response is not mounted in these hosts (21). In that study, *Brucella* CFU counts were determined on a weekly basis, but CFU kinetics and immune response within the first days of infection were not addressed. The aim of the present study was to characterize the innate immune response in the lungs of mice, and the bacterial dissemination to peripheral organs, during the first days after intratracheal infection with *B. abortus*. A potential role of Btps in the modulation of the lung immune response to the infection and to the stimulation with TLR agonists was also investigated.

## Materials and Methods

### Animals

Male 7- to 8-week-old Balb/c mice were used in all experiments. The animals were housed in BSL3 facilities (IIB-INTECH-UNSAM, Buenos Aires, Argentina). Animals were housed in groups of five animals, under controlled temperature (22 ± 2°C) and artificial light under a 12-h cycle period. All experimental protocols of this study were conducted in agreement with international ethical standards for animal experimentation (Helsinki Declaration and its amendments, Amsterdam Protocol of welfare and animal protection, and National Institutes of Health, USA, guidelines: Guide for the Care and Use of Laboratory Animals). The protocols of this study were approved by the Institutional Committee for the Care and Use of Experimentation Animals from the University of San Martin (UNSAM).

### Bacterial Strains and Growth Conditions

*Brucella abortus* 2308 (wild-type strain, WT) and its isogenic *B. abortus btpAbtpB* double mutant (9) were grown in tryptic soy broth at 37°C with agitation until reaching an approximate OD_600_ of 1.0. Bacteria were washed twice with sterile phosphate-buffered saline (PBS), and inocula were prepared in sterile PBS on the basis of the optical density readings, but the actual concentration was later checked by plating on tryptic soy agar (TSA). All live *Brucella* manipulations were performed in biosafety level 3 facilities.

### Intratracheal Inoculation

Animals were inoculated intratracheally with *B. abortus* WT or *B. abortus btpAbtpB* mutant as previously described (22) with minor modifications. Briefly, animals were anesthetized with isoflurane, and after becoming recumbent, were injected intraperitoneally with a mixture of ketamine and xylazine (100 and 8 mg/kg). Mice were placed in supine position over an acrylic backboard and restrained by the teeth using a rubber band. Under translucent illumination of the trachea, the inoculum was injected in a final volume of 20 µl in between the vocal cords with a Hamilton syringe coupled to a blunt-ended probe, to deliver 10^6^ CFU per mice. Control mice received 20 µl of PBS following the same procedure.

### CFU and Cytokine Analysis

At different time points post-infection (p.i.), mice were euthanized by an intraperitoneal injection of a lethal dose of ketamine and xylazine, and their lungs, liver, and spleens were harvested. The whole organs were homogenized in 2 ml sterile PBS, and serial dilutions of homogenate aliquots were plated on TSA for CFU counting. The remaining homogenate volumes were centrifuged for 15 min, and the supernatants were mixed with protease inhibitors (cOmplete™, Roche) and stored at −70°C for cytokine and chemokine determination by commercial ELISA kits (R&D), according to the manufacturer’s instructions.

### Bronchoalveolar Lavage Fluid (BALF)

Mice were euthanized, their tracheas were cannulated, and the airways were perfused several times with 0.7 ml of sterile cold PBS containing 1 mM EDTA to provide 4 ml of BALF. BALF samples were centrifuged at 400 × *g* for 10 min at 4°C and supernatants were stored at −70°C for cytokine measurements as described above.

### Histological Examination

At 2 and 7 days p.i., lungs were harvested and fixed in 4% paraformaldehyde for 24 h, then embedded in paraffin and cut in 5-µm sections. The samples were stained with hematoxylin and eosin and blindly analyzed by a pathologist. Tissue damage was graded using a previously described scoring system (23), as follows: 0 (normal = no inflammation), 1 (minimal = perivascular, peribronchial, or patchy interstitial inflammation involving less than 10% of lung volume), 2 (mild = perivascular, peribronchial, or patchy interstitial inflammation involving 10–20% of lung volume), 3 (moderate = perivascular, peribronchial, patchy interstitial, or diffuse inflammation involving 20–50% of lung volume), and 4 (severe = diffuse inflammation involving more than 50% of lung volume).

### Inhibition of LPS-Induced Airway Inflammation

Mice were intratracheally inoculated with PBS or with 10^6^ CFU/mouse of either *B. abortus* WT or *B. abortus btpAbtpB* mutant as described above, and 24 h later were intratracheally inoculated with 5 µg of *E. coli* LPS (Sigma-Aldrich). Lungs were harvested 24 h later, fixed in paraformaldehyde 4%, and subjected to histological analysis as described above.

### Pulmonary Innate Immune Response Stimulation

Mice were intratracheally inoculated with 5 µg of *E. coli* LPS and 24 h later were intratracheally inoculated with PBS or with 10^6^ CFU of either *B. abortus* WT or *B. abortus btpAbtpB* mutant. Lungs were harvested 24 h later, homogenized, and analyzed for cytokines and CFU counts.

### Statistical Analysis

Statistical comparisons for significant differences were performed with the ANOVA test followed by Tukey’s test or Dunnett’s test using GraphPad 5.0 software. Normality was assessed by the D’Agostino–Pearson test. Data are means ± SEM from at least three independent experiments. A *p* value <0.05 was considered as statistically significant.

## Results

### Kinetics of *Brucella* Infection in Lungs and Dissemination to Peripheral Organs

**Figure 1:**
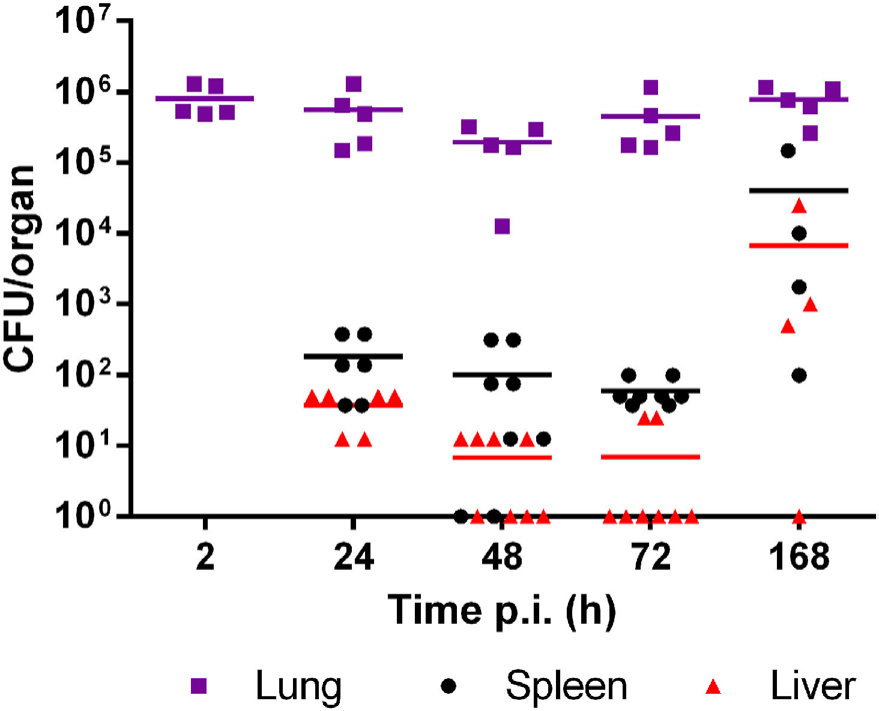
*Brucella abortus* persists in lungs but disseminates to peripheral organs in the first week after intratracheal infection. Mice were infected with 10^6^ CFU/mouse of *B. abortus* WT, and CFU numbers were determined in lung, liver, and spleen homogenates at different times post-infection (p.i.) (*n* = 5–8 per time point).

Mice were intratracheally infected with *B. abortus* WT, and CFU numbers were determined in lung, liver, and spleen homogenates at different times within the first week p.i. As shown in Figure 1, the pulmonary bacterial burden diminished non-significantly during the first days p.i. but then rapidly reached values similar to the initial inoculum. *Brucella* could disseminate from the initial infection site and was recovered as early as 1 day p.i. from spleen and liver. The bacterial load in liver and spleen tended to increase at 7 days (168 h) p.i. as compared to previous days, although the difference did not reach statistical significance probably due to data dispersion (Dunnett’s test versus 24 h p.i.).

### *B. abortus* Induces a Mild Inflammatory Response in Murine Lungs

**Figure 2:**
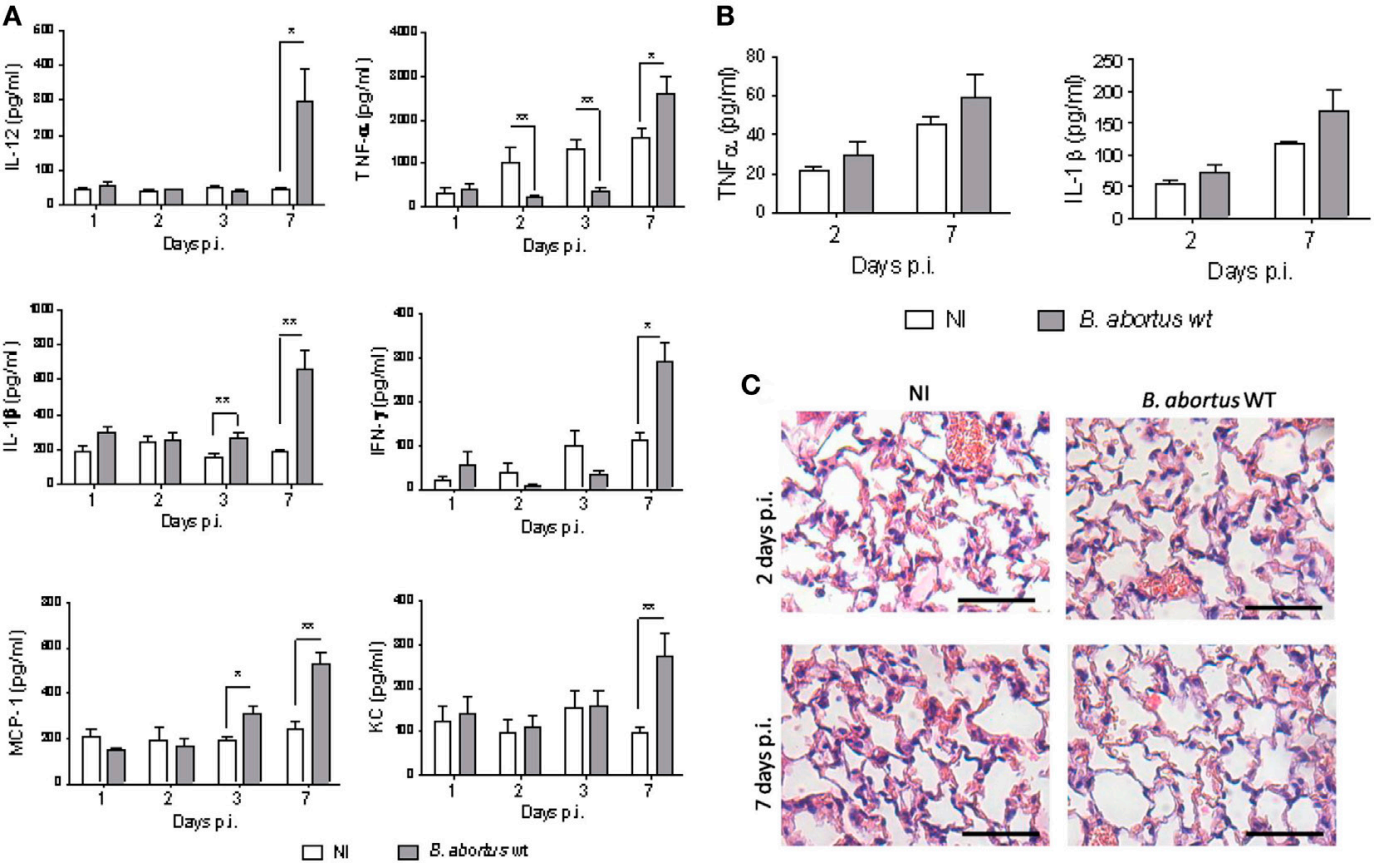
*Brucella abortus* induces a delayed and mild inflammatory response. Mice (*n* = 5) were infected intratracheally with 10^6^ CFU of *B. abortus* (gray bars) and cytokines were measured at different times post-infection (p.i.) in lung homogenates **(A)** and bronchoalveolar lavage fluid (BALF) **(B)**. Controls inoculated with phosphate-buffered saline through the same route (white bars) were assessed in parallel. Differences between groups were analyzed by ANOVA followed by Tukey’s test (*p < 0.05, **p < 0.01). Histological changes in lungs were assessed in infected and control mice at 2 and 7 days post-inoculation **(C)**. Magnification, ×20. Bar = 50 µm.

To assess the early pulmonary cytokine response induced by the intratracheal infection with *B. abortus* WT, levels of IL-1β, TNF-α, IFN-γ, IL-12, MCP-1, and KC were measured in lung homogenates from infected mice. A significant increase in IL-1β and MCP-1 concentration was found at 3 days p.i. as compared to non-infected mice, whereas a significant increase in IL-12, KC, TNF-α, and IFN-γ was detected only at 7 days p.i. (Figure 2A). Due to sample volume limitations, only TNF-α and IL-1β could be determined in BALF. Both cytokines tended to be higher in BALF from infected mice than in control mice, but differences did not reach significant differences (Figure 2B). Overall, these results suggest a delayed cytokine response to *B. abortus* in the lungs of mice infected through the intratracheal route.

Cellular infiltrates are commonly found in lungs as components of the innate immune response to infection by inhaled pathogens. Therefore, we assessed histological changes in lungs of mice infected with *B. abortus* WT, as compared to non-infected controls. As shown in Figure 2C, at 2 days p.i., no peribronchial or perivascular infiltrates were present in infected mice, and only a mild and focalized lymphocytic interstitial infiltrate and a few points of hematic extravasation were noticed. The interstitial infiltrate was milder at day 7 p.i., and some hematic extravasation foci were also observed.

### *Brucella* Actively Suppresses TLR4-Mediated Lung Inflammation

The limited inflammatory response elicited by *B. abortus* in the lungs of infected mice may be due to some of the immune evasion mechanisms of *Brucella*, such as the secretion of proteins that interfere with TLR signaling thus downmodulating cytokine production (24, 25). Therefore, experiments were conducted to evaluate if *B. abortus* was capable to actively suppress lung inflammation caused by *E. coli* LPS, a potent TLR4 agonist. Mice were infected intratracheally with *B. abortus*, or administered with PBS as a control, and 24 h later received *E. coli* LPS by the intratracheal route. The following day mice were euthanized, and their lungs were harvested for cytokine measurement and histological examination.

**Figure 3:**
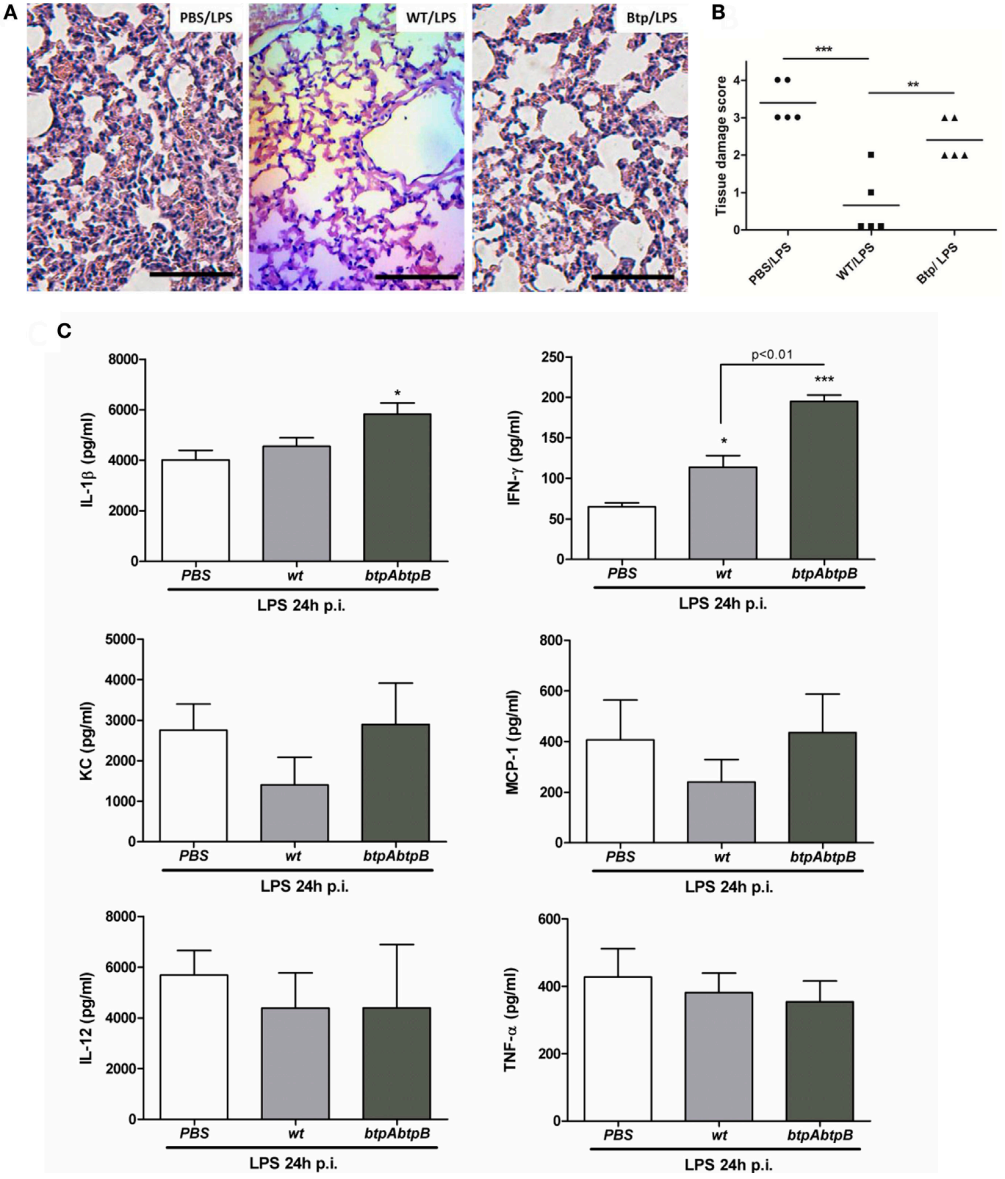
*Brucella* preinfection suppresses TLR4-mediated lung inflammation. Mice (*n* = 5) were infected intratracheally with 10^6^ CFU of *Brucella abortus* 2308 (WT) or a double mutant for BtpA and BtpB proteins (*btpAbptB*), or were inoculated with phosphate-buffered saline (PBS) as a control, and 24 h later received *E. coli* LPS by the intratracheal route. The next day mice were euthanized and their lungs were harvested for histological examination **(A,B)** and for preparing homogenates for cytokine measurement **(C)**. **(B)** **p < 0.001, ***p < 0.001, Tukey’s multiple comparison test; **(C)** *p < 0.05, ***p < 0.001 vs. the PBS/LPS group. Magnification, ×20. Bar = 50 µm.

As shown in Figure 3A (left panel), LPS inoculation to mice previously administered with PBS (PBS/LPS group) causes lung inflammation, as expected for a TLR4 agonist. Lungs exhibited vascular congestion, edema, and focal points of hematic extravasation in the alveoli, along with an important infiltrate of polymorphonuclear cells. This inflammatory reaction was markedly abolished in mice preinfected with *B. abortus* WT (WT/LPS) as only mild vascular congestion and edema were observed (Figure 3A, middle panel). As shown in Figure 3B, the inflammatory score found in lungs from the WT/LPS group was significantly lower than that found in the PBS/LPS group. These results show that *B. abortus* modulates the pulmonary innate immune response to a TLR4 agonist.

As part of the wide repertoire of *Brucella* immune evading effectors, two proteins have been described, namely, *Brucella* TIR-containing proteins A (BtpA) and B (BtpB), which interfere with TLR signaling and innate immune responses (9, 10). To test whether these proteins are involved in the modulation of LPS-mediated inflammation, mice were intratracheally infected with a *B. abortus* double mutant for the Btp proteins (*B. abortus btpAbtpB*), and 24 h later were inoculated with *E. coli* LPS through the same route.

As compared to the lungs of mice preinfected with the WT strain (WT/LPS), those of mice infected with the double mutant (*btpAbtpB*/LPS) exhibited an important polymorphonuclear infiltrate and some hematic extravasation focal points (Figure 3A, right panel). Nevertheless, the polymorphonuclear infiltrate of the *btpAbtpB*/LPS group was still milder than that of the PBS/LPS group. Lungs from both WT/LPS and *btpAbtpB*/LPS groups had less vasocongestion than those from the PBS/LPS group. As shown in Figure 3B, the inflammatory score found in lungs from the *btpAbtpB*/LPS group was significantly higher than that found in the WT/LPS group, but lower than that in the PBS/LPS group. Altogether, these results indicate that the immunosuppression observed in mice from WT/LPS group is partly due to the Btp proteins of *Brucella*.

The modulatory effect of Btp proteins in this model was also evidenced at the cytokine level (Figure 3C). The levels of IFN-γ in lung homogenates were significantly lower in mice from the WT/LPS group than in those from the *btpAbtpB*/LPS group, and a similar tendency was observed for IL-1β, KC, and MCP-1 levels. In agreement with the reduced inflammatory infiltrate in the WT/LPS group versus the PBS/LPS group, levels of the KC and MCP-1 chemokines tended to be lower in the first group.

### Btp Proteins Modulate the Lung Inflammatory Response to *B. abortus*

**Figure 4:**
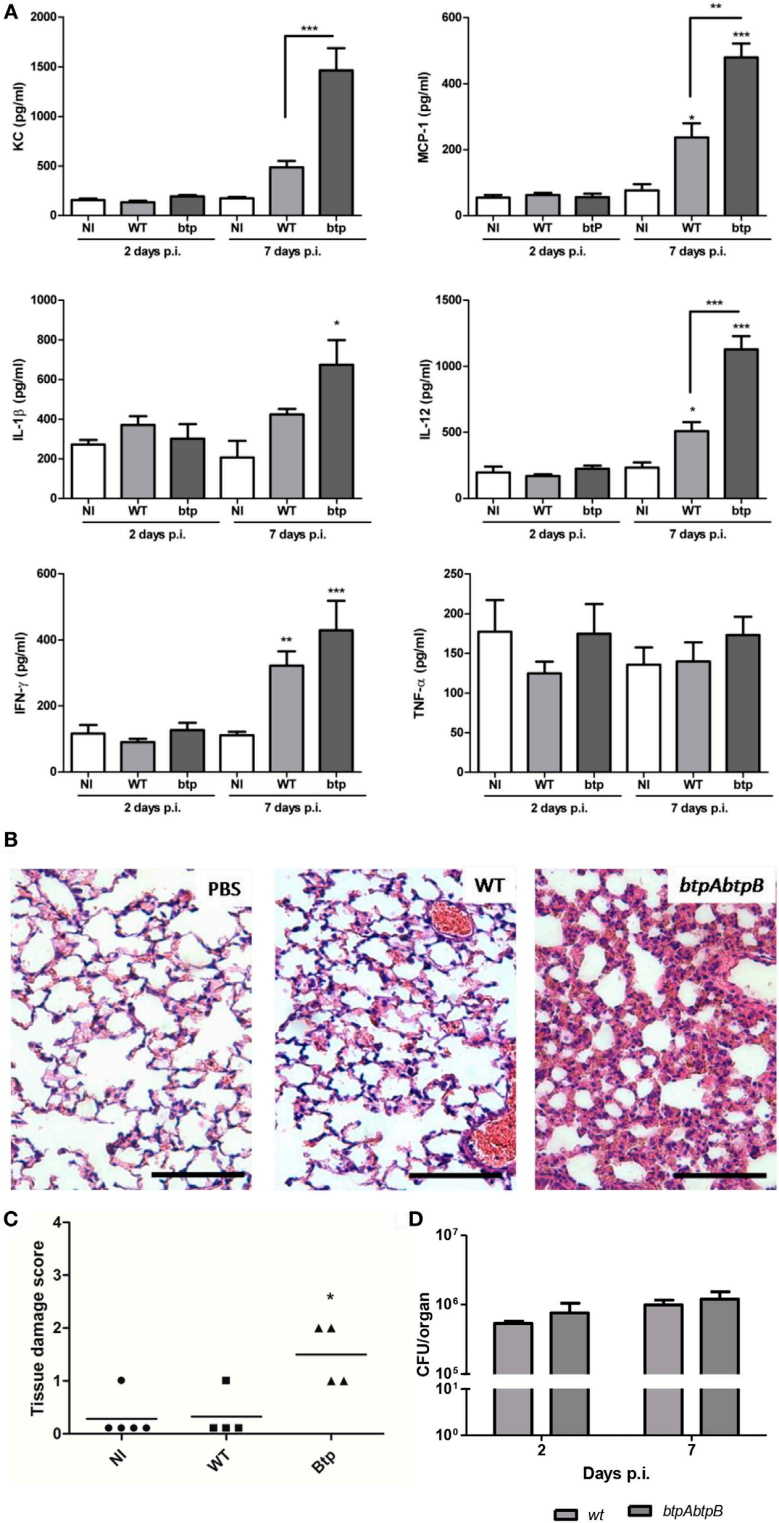
Btp proteins modulate the lung inflammatory response to *Brucella abortus*. Mice (*n* = 5) were infected intratracheally with 10^6^ CFU of either *B. abortus* WT or *B. abortus btpAbtpB* mutant, or received phosphate-buffered saline (PBS) through the same route as a control. At 2 and 7 days post-infection (p.i.), animals were sacrificed and their lungs were obtained to evaluate cytokine response **(A)**, histology **(B,C)**, and colony-forming units **(D)**. *p < 0.05, **p < 0.01, ***p < 0.001 versus NI. Magnification, ×20. Bar = 50 µm.

The role of Btp proteins in the course of a *Brucella* infection acquired through a natural route is still unknown. We hypothesized that Btp proteins could contribute to modulate the innate response in lungs during a respiratory infection with *Brucella*. To test this hypothesis, we evaluated the pulmonary histology and cytokine response of mice infected intratracheally with either *B. abortus* WT or the *B. abortus btpAbtpB* mutant. The cytokine analysis revealed no significant differences in proinflammatory cytokines (IL-1β, IFN-γ, IL-12, and TNF-α) and chemokines (KC and MCP-1) at 2 days p.i. However, at day 7 p.i., there was a significant increase of IL-12, KC, and MCP-1 in *btpAbtpB*-infected lungs when compared with WT-infected lungs (Figure 4A), and a similar but non-significant tendency was observed for IL-1β and IFN-γ. Lungs from *btpAbtpB^−^* infected mice presented a higher level of inflammatory infiltrate than those from WT-infected mice at both 2 days p.i. (Figures 4B,C) and 7 days p.i. (not shown). Despite this stronger proinflammatory profile in lungs of mice infected with the Btp mutant, no significant differences in CFU counts were observed in comparison with WT infection at 2 or 7 days p.i. (Figure 4D).

### Btp Proteins Protect *B. abortus* from TLR4-Induced Lung Inflammation

**Figure 5:**
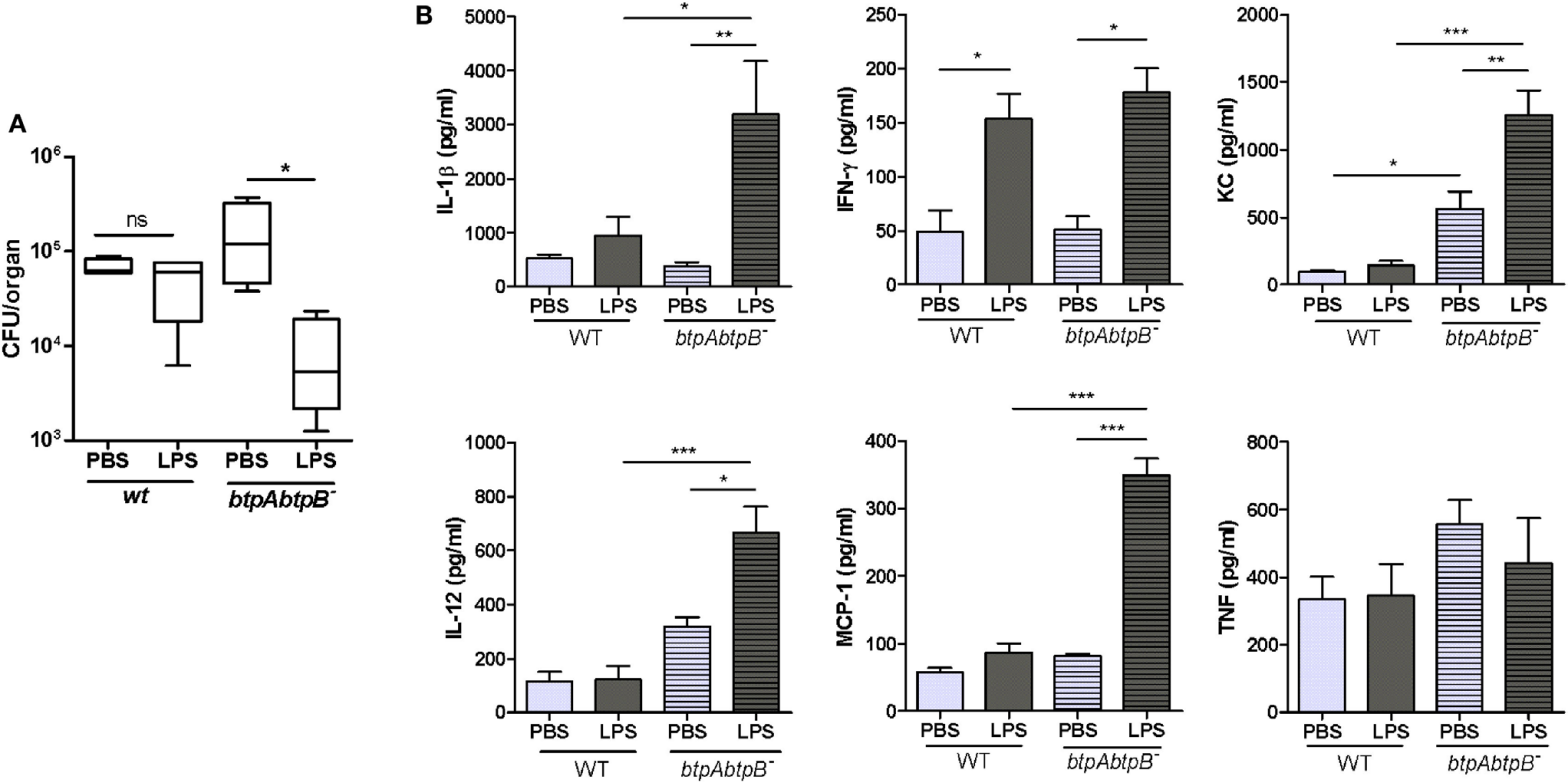
Btp proteins protect *Brucella abortus* from TLR4-induced lung inflammation. Mice (*n* = 5) were administered with an intratracheal dose of *E. coli* LPS (5 μg/mouse) and one day post-administration were inoculated with phosphate-buffered saline (PBS), *B. abortus* WT or *B. abortus btpAbtpB* mutant. The day after infection, lungs of the different groups were collected and analyzed for CFU **(A)** and cytokines **(B)** (*p < 0.05, **p < 0.01, ***p < 0.001).

As shown above, despite the increased levels of cytokines and the increased inflammatory infiltrate in the lungs of *btpAbtpB*-infected mice as compared to WT-infected controls, CFU counts of the mutant did not change during the first week of infection. This suggests that the defensive mechanisms mounted during the first stages of infection are not enough to reduce the pulmonary load of *Brucella*, even if the bacterium is devoid of Btp proteins. To test whether a stronger pulmonary innate immune response could reduce the number of *Brucella* CFU in lungs, mice were administered with an intratracheal dose of *E. coli* LPS (5 μg/mouse) and 1 day post-administration were inoculated through the same route with PBS, *B. abortus* WT, or *B. abortus btpAbtpB*. The day after infection, lungs of the different groups were collected and analyzed for cytokines and CFU. At 24 h p.i. (48 h after LPS treatment), the pulmonary levels of several proinflammatory cytokines (IL-1β, IL-12, MCP-1, and KC) were significantly higher in the LPS/*btpAbtpB* group than in the LPS/WT group, suggesting that Btp proteins modulate the proinflammatory response elicited by the LPS pretreatment (Figure 5B). At this time point, the cytokine response to WT infection did not differ significantly between PBS- and LPS-pretreated mice, except for IFN-γ which was significantly increased in LPS-pretreated animals. By contrast, among animals infected with the *btpAbtpB* mutant, the levels of all cytokines, except for TNF-α, were significantly higher in LPS-conditioned lungs than in those pretreated with PBS. As shown in Figure 5A, there was no significant difference in CFU counts between lungs from WT*-*infected mice previously treated with either LPS or PBS. Among mice infected with the *btpAbtpB* mutant, by contrast, CFU numbers were significantly reduced in LPS-conditioned lungs as compared to those pretreated with PBS. Globally, these results suggest that Btp proteins play a role in *Brucella* survival within a strong inflammatory environment.

## Discussion

The airborne route of infection has been widely shown to be epidemiologically important in human and animal brucellosis. After entry through inhalation, bacteria of the *Brucella* genus can disseminate from the lungs to the rest of the organism. However, pulmonary manifestations have been only exceptionally reported in patients who acquired *Brucella* infection through inhalation of contaminated aerosols (4). This should not be confused with cases of pulmonary brucellosis (including pneumonia and pleural effusion), which have been described in some series on human brucellosis. In these series, consumption of unpasteurized dairy products was the most common source of infection, strongly suggesting that *Brucella* had reached the lungs through hematogenous dissemination (26–28). The scarcity of pulmonary manifestations in patients with airborne-acquired brucellosis suggests that, in spite of the high infectivity of *Brucella* through inhalation, an inflammatory immune response against the pathogen is mild or nearly absent in the lungs.

In this study, we demonstrated that *B. abortus* intratracheal infection produces a mild proinflammatory response in lungs of infected Balb/C mice partly due to immune modulation. The cytokine response and the histological analysis in lungs showed minimal alterations in infected animals, and such changes started as late as 3 days p.i. This lack of vigorous inflammatory response may partially explain the steady CFU counts of *B. abortus* in murine lungs during the 1-week follow-up of the present study. Notably, a longer bacteriological follow-up after infection with aerosolized *B. abortus* found steady pulmonary CFU counts for up to 8 weeks p.i (21). In line with our findings, a recent study on intranasal infection with *B. melitensis* in mice also reported invariable levels of CFU during the first 12 days p.i (29). Although the later study used a different *Brucella* species and a different delivery method as compared to our study, it also revealed a lack of significant inflammatory response in the lungs of infected mice. However, the mechanisms underlying this lack of inflammation were not explored.

The limited inflammatory response elicited by *B. abortus* in the lungs of infected mice may be due to some of the immune evasion mechanisms of *Brucella*, which include poor immunostimulating antigens and active suppression mechanisms (9, 24, 29). Among the latter, the action of Btp proteins, which contain TIR motifs that interact with TIR-containing components of the TLR signaling cascade, is especially remarkable (24, 25). Btp proteins have been shown to downmodulate the secretion of proinflammatory cytokines by *Brucella*-infected dendritic cells, and to reduce the formation of splenic granulomas in infected mice. Therefore, the interference with TLR signaling through the action of Btp proteins was considered a plausible mechanism for the mild inflammatory response to *B. abortus* in our model. Experiments were conducted to evaluate if *B. abortus* was capable of actively suppressing lung inflammation caused by *E. coli* LPS, a potent TLR4 agonist. *B. abortus* preinfection not only inhibited the LPS-induced recruitment of phagocytes to the lungs but also reduced the pulmonary production of some proinflammatory cytokines in response to LPS. Notably, when mice were preinfected with the *B. abortus btpAbtpB* double mutant, this inhibitory effect on LPS-induced inflammation was partially lost, as revealed by histology analysis and KC measurement. This suggests that Btps are partially responsible for the inhibitory effect, although additional immune evasion mechanisms may be also involved in the ability of *B. abortus* to inhibit LPS-induced pulmonary inflammation. The btpAbtpB/LPS group presented less inflammatory infiltrate than the PBS/LPS group in spite of higher levels of IL-1β and IFN-γ. The reduced inflammatory infiltrate may not be due to differences in the chemokines measured (MCP-1 and KC), which were similar in both groups, suggesting that other cytokines not measured in the present study may be involved.

Btp proteins also seemed to exert immunomodulation in the context of pulmonary *B. abortus* infection, as a greater degree of lung inflammation was detected in mice intratracheally infected with the *btpAbtpB* mutant than in those infected with the WT strain. However, this increased inflammation did not translate into an enhanced infection control, as there were no differences at 2 or 7 days p.i. in the pulmonary CFU counts of the mutant as compared to the WT strain. These findings suggest that, regardless of the expression of Btp proteins, the immune response mounted in the lung during the first stages of infection is not enough to reduce the pulmonary load of *Brucella*. In line with these findings, a recent study has also suggested that the pulmonary inflammatory response is irrelevant for the early control of *B. melitensis* after intranasal infection, as pulmonary CFU counts did not differ between wild-type mice and those deficient for IL-1R, IL-6, TNF-α, or CCR2 (29).

Experiments were carried out in the present study to test whether a stronger innate immune response in the lungs could reduce the pulmonary CFU counts of *Brucella*, and whether Btps may have a role in *Brucella* survival in this enhanced inflammatory environment. For this, mice were intratracheally inoculated with *E. coli* LPS before infection with *B. abortus* WT or *B. abortus btpAbtpB*. The pulmonary levels of several proinflammatory cytokines were significantly higher in mice of the LPS/*btpAbtpB* group than in those from the LPS/WT group, suggesting that Btp proteins modulate the proinflammatory response elicited by the LPS pretreatment. Whereas LPS pretreatment did not modify the survival of *B. abortus* WT, it reduced the survival of the *btpAbtpB* mutant. The overall results suggest that the expression of Btp proteins does not confer a survival advantage to *B. abortus* in the context of the weak inflammatory environment elicited in the lungs by the infection with this pathogen, but may confer such advantage within a stronger inflammatory environment. Further studies will be required to address this question in different models of *Brucella* infection *in vivo*. To our best knowledge, this is the first study to address the role of Btp proteins in mice infected through a natural route for *Brucella* species, as previous studies used intraperitoneal infection (9, 30).

Overall, this study revealed a weak proinflammatory response to inhaled *B. abortus* in murine lungs. In addition, the cytokine response started to differentiate from the control group as late as 3 days p.i., 2 days after *Brucella* was first detected in peripheral organs. This means that *Brucella* can disseminate systemically before an inflammatory response is achieved in the lungs. In the present study, *B. abortus* was found in spleen and liver as early as 1 day after intratracheal infection. These findings are in line with those of Archambaud et al., who found the bacterium in mediastinal lymph nodes as soon as 1.5 days after intranasal infection (31). According to that report, the bacterium seems to be transported out of the lungs by alveolar macrophages and, a bit later, by dendritic cells. The present study also suggests that the pulmonary inflammatory response mounted at later time points has only a limited impact on *B. abortus* survival in the lungs, as CFU counts at 7 days p.i. did not decline as compared to the initial inoculum. The limited antimicrobial action of the pulmonary innate immunity against *Brucella* infection may also contribute to the efficiency of this pathogen for producing systemic disease after inhalation, as the persistent pulmonary bacterial pool may constitute a continuous source for dissemination to peripheral organs.

In summary, this study shows that *B. abortus* induces only a weak inflammatory response in lungs after intratracheal infection. Whereas the lack of a stronger inflammation is explained in part by the modulating effect of Btp proteins on TLR-mediated responses, the increased inflammatory response elicited in the absence of this modulation is still insufficient for controlling *B. abortus* infection in the lungs. The limited antimicrobial action of the pulmonary innate immunity against *Brucella* infection may contribute to the efficiency of this pathogen to produce systemic disease after inhalation.

## Ethics Statement

All experimental protocols of this study were conducted in agreement with international ethical standards for animal experimentation (Helsinki Declaration and its amendments, Amsterdam Protocol of welfare and animal protection, and National Institutes of Health, USA, guidelines: Guide for the Care and Use of Laboratory Animals). The protocols of this study were approved by the Institutional Committee for the Care and Use of Experimentation Animals from the University of San Martin (UNSAM).

## Author Contributions

Conceived and designed the experiments: MH, MF, and PB. Performed the experiments: MH, MF, AF, and JF. Analyzed the data: MH, MF, AF, JF, SV, DC, and PB. Wrote the draft and/or final version paper: MH, MF, and PB.

## Conflict of Interest Statement

The authors declare that the research was conducted in the absence of any commercial or financial relationships that could be construed as a potential conflict of interest.
